# Genome-wide DNA N6-methyladenosine in *Aeromonas veronii* and *Helicobacter pylori*

**DOI:** 10.1186/s12864-024-10074-y

**Published:** 2024-02-08

**Authors:** Honghao Zhao, Jiayue Ma, Yanqiong Tang, Xiang Ma, Juanjuan Li, Hong Li, Zhu Liu

**Affiliations:** School of Life and Health Sciences, Hainan University, Haikou, China

**Keywords:** DNA N6-methyladenosine modification, Single-molecule real-time sequencing, *Aeromonas veronii*, Gene expression

## Abstract

**Background:**

DNA N6-methyladenosine (6mA), as an important epigenetic modification, widely exists in bacterial genomes and participates in the regulation of toxicity, antibiotic resistance, and antioxidant. With the continuous development of sequencing technology, more 6mA sites have been identified in bacterial genomes, but few studies have focused on the distribution characteristics of 6mA at the whole-genome level and its association with gene expression and function.

**Results:**

This study conducted an in-depth analysis of the 6mA in the genomes of two pathogenic bacteria, *Aeromonas veronii* and *Helicobacter pylori*. The results showed that the 6mA was widely distributed in both strains. In *A. veronii*, 6mA sites were enriched at 3’ end of protein-coding genes, exhibiting a certain inhibitory effect on gene expression. Genes with low 6mA density were associated with cell motility. While in *H. pylori*, 6mA sites were enriched at 5’ end of protein-coding genes, potentially enhancing gene expression. Genes with low 6mA density were closely related to defense mechanism.

**Conclusions:**

This study elucidated the distribution characteristics of 6mA in *A. veronii* and *H. pylori*, highlighting the effects of 6mA on gene expression and function. These findings provide valuable insights into the epigenetic regulation and functional characteristics of *A. veronii* and *H. pylori*.

**Supplementary Information:**

The online version contains supplementary material available at 10.1186/s12864-024-10074-y.

## Background

DNA N6-methyladenosine (6mA) refers to the addition of a methylation modification located on the 6th nitrogen atom of adenine by specific DNA adenine methyltransferases, which is an important epigenetic mark in biological inheritance [1]. 6mA was first discovered in *Escherichia coli* in the 1950s [2, 3], and subsequently found in other bacteria [4]. Blow et al. found that over 90% of organisms have 6mA after studying more than 200 bacteria and archaea [5]. 6mA plays an important role in physiological processes in microorganisms, including replication initiation [6], mismatch repair [7], transcription, and also has a significant impact on virulence, antibiotic resistance, and antioxidant abilities [8, 9]. Therefore, it has become another focus in research on microbial drug resistance, stress response, and pathogenicity, as well as a hot topic in the study of microbial interactions with the environment. However, few studies have researched on the distribution patterns of 6mA across the entire bacterial genome and its association with gene expression and function.

Currently, techniques to identify 6mA mainly fall into two categories. The first category is quantification of methylated nucleotide, including dot blot and liquid chromatography coupled with tandem mass spectrometry (LC–MS) [10, 11]. They infer the degree of 6mA by detecting the ratio of 6mA-modified adenine to total adenine in DNA [12]. The disadvantage of this category of technique is that it cannot provide information on the location of 6mA. The second category is high-throughput sequencing techniques, including 6mA-IP-seq and single molecule real time (SMRT) sequencing. 6mA-IP-seq uses an antibody that binds to DNA fragments with 6mA for sequencing, enabling the localization of 6mA within a range of tens of base pairs. However, its accuracy in 6mA detection remains to be improved. SMRT sequencing is based on variances in interpulse duration (IPD) between two successive base incorporations in modified sites of DNA template. It is a powerful technique for detection of 6mA at single-nucleotide resolution and single-molecule level [13]. It has the advantages of high accuracy, broad research scope and cost-effectiveness. SMRT sequencing has provided insights into 6mA and revealed that 6mA is widely present in many bacteria, such as *Neisseria meningitidis* [14], *Geobacter metallireducens* [15], and *Campylobacter coli* [16].

Proteobacteria is the largest phyla and contains many pathogenic bacteria. Based on rRNA sequences, Proteobacteria can be divided into five classes: Alphaproteobacteria, Betaproteobacteria, Gammaproteobacteria, Deltaproteobacteria, and Epsilonproteobacteria[17]. In recent years, there has been an increase in infections caused by *Aeromonas veronii* belonging to Gammaproteobacteria and *Helicobacter pylori* belonging to Epsilonproteobacteria. *A. veronii*, can not only infect aquatic organisms such as grass turtles, rosemary shrimps and Siamese crocodiles but also mammals including humans, causing gastroenteritis, peritonitis, meningitis, and septicaemia, posing a great threat to aquaculture and human health [18]. *H. pylori* is a microaerophilic parasite that resides in the human gastric mucosa, which is closely related to gastrointestinal diseases such as chronic gastritis, peptic ulcers, intestinal gastric cancer, as well as various extra-gastrointestinal diseases such as unexplained iron-deficiency anemia, idiopathic thrombocytopenic purpura, etc. [19]. If the 6mA in *A. veronii* and *H. pylori* could be detected, the distribution patterns of 6mA at the whole-genome scale could be analyzed to determine associations between 6mA and gene expression and function. This will provides a solid theoretical basis for understanding the epigenetic regulation mechanisms of *A. veronii* and *H. pylori*, and new ideas for pathogenesis research.

Therefore, this study first used PacBio SMRT sequencing to detect the high-quality 6mA sites in *A. veronii* and *H. pylori*. Further, we analyzed the distribution characteristics of 6mA in protein-coding genes and non-coding RNA genes, and explored the effects of different levels of 6mA on gene expression and function. The results showed that 6mA tended to occur at 3’ end of protein-coding genes in *A. veronii*, which was related to cell motility. In *H. pylori*, 6mA exhibited a preference at 5’ end of protein-coding genes, which was associated with defense mechanism. In both strains, 6mA appeared a propensity for occurrence in rRNA genes compared to tRNA or sRNA genes. Moreover, 6mA exerted a significant promoting effect on gene expression in *H. pylori*. These results undoubtedly broaden our understanding of *A. veronii* and *H. pylori*.

## Results

### Identification of 6 mA in *A. veronii* genome

**Fig. 1:**
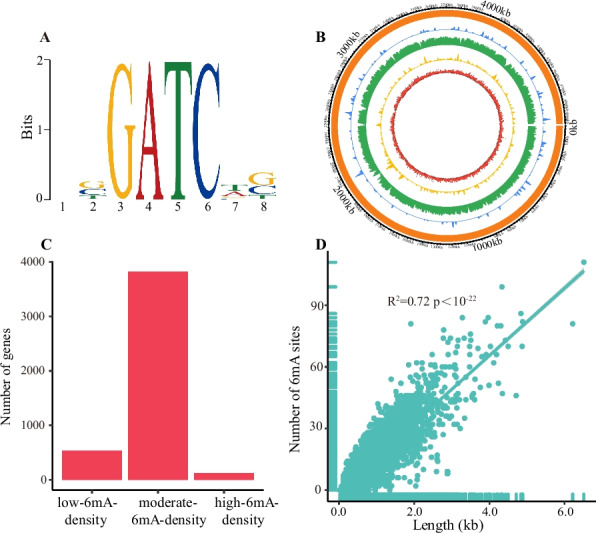
Identification of 6mA in *Aeromonas veronii* genome. **A** The motif enriched with 6mA. Information content at a motif position, where all nucleoside with equal probability, is 0 bits, while a position with exclusive occurrence of a single nucleotide had an information content of 2 bits. **B** Circos plot illustrating the genomic distribution of 6mA. Blue, green and yellow rings represented the regions with high, moderate and low 6mA levels, respectively. Red ring represented gene expression level. **C** Number of genes with different 6mA levels. The genes were categorized as low-6mA-density, moderate-6mA-density and high-6mA-density genes according to their 6mA density. **D** Correlation between the number of 6mA sites and the gene length

A total of 74,601 6mA sites were identified in the genome of *A. veronii* based on SMRT sequencing data, with comparable numbers on both the positive and negative strands (Table 1). The 6mA density (6mA/A) in the genome was calculated to be 3.65% (Table 1).To explore whether 6mA occurs in specific sequences, we searched for motifs significantly enriched with 6mA. The GATC motif (E-value < 1.00 × 10^–22^; Fig. 1A) was detected. A total of 34,382 (99.94%) and 34,339 (99.81%) A bases within the GATC motif were methylated on the positive and negative strands, respectively. To pinpoint the methyltransferase responsible for modifying the GATC motif in *A. veronii*, all high-quality GATC-specific DNA methyltransferases were collected and executed to construct a Hidden Markov Model (HMM). By aligning with the HMM, the GATC-specific DNA methyltransferase (GenBank accession number: UZE58634) was obtained in *A. veronii*.

**Table 1:** N6-menthyladenine (6mA) density across the genomic DNA

Species	Genome size (bp)	Number of A base on the forward strand	Number of A base on the reverse strand	Number of A base	Number of 6mA on the forward strand	Number of 6mA on the reverse strand	Number of 6mA	Density on the forward strand	Density on the reverse strand	Density
*Aeromonas veronii*	4,926,096	1,021,715	1,022,614	2,044,329	37,321	37,280	74,601	3.65%	3.65%	3.65%
*Helicobacter pylori*	1,667,876	505,397	514,075	1,019,472	24,200	25,086	49,286	4.79%	4.88%	4.83%

### Distribution of 6 mA in *A. veronii* genome

**Fig. 2:**
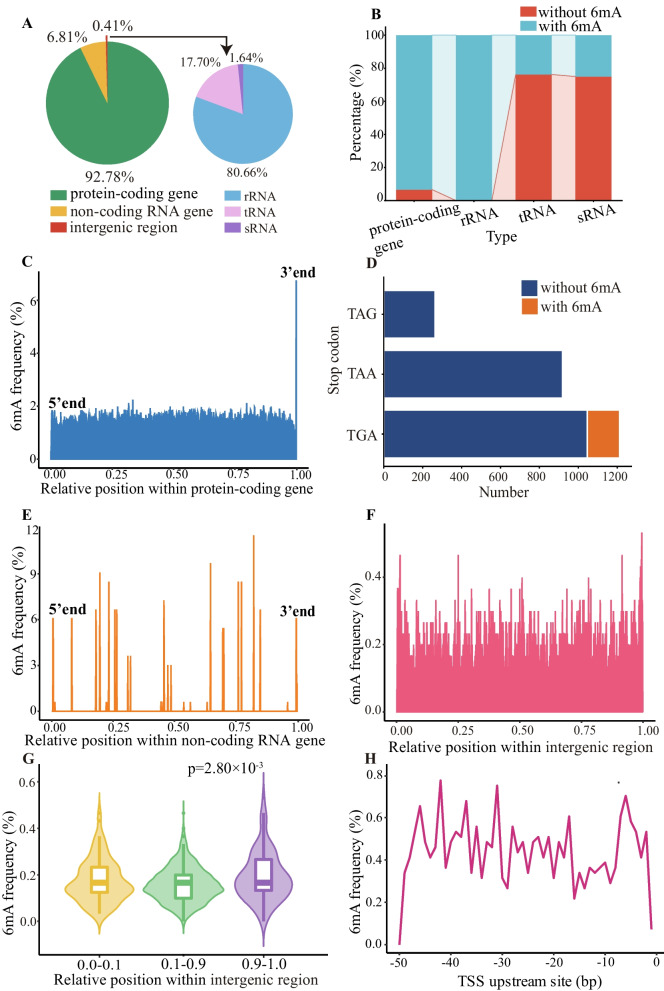
Distribution of 6mA in *A. veronii* genome. **A** Percentage of 6mA sites across different types of genes. Each color corresponded to a specific gene type. **B** Percentage of genes with and without 6mA. **C** Frequency of 6mA sites at each relative position within protein-coding genes. **D** Number of stop codons with and without 6mA. **E** Frequency of 6mA sites at each relative position within non-coding RNA genes. **F** Frequency of 6mA sites at each relative position within intergenic regions. In (C), (E), and (F), if a gene/intergenic region had N bases, the relative position of the base at the i-th position was represented as i/N, ranging between 0 and 1. **G** Comparison of 6mA frequencies in different relative segments within intergenic regions. The relative positions (0–1) within the intergenic region were divided into three segments, 0.0–0.1, 0.1–0.9 and 0.9–1.0. The bases in the 0.0–0.1 and 0.9–1.0 segments were in close proximity to the gene body. *P*-values were calculated by Wilcoxon test. No significant difference in 6mA frequency was observed between the 0.0–0.1 and 0.1–0.9 segments (*P* value > 0.01). **H** Frequency of 6mA sites at each position in the upstream 50 bp of transcription start site (TSS)

*A. veronii* had 4724 protein-coding genes and 165 non-coding RNA genes. 6mA level at each gene across the genomic DNA was profiled. According to this, genes were classified into high-6mA-density genes, moderate-6mA-density genes, and low-6mA-density genes. The result showed that the majority of genes belonged to the moderate-6mA-density genes, indicating a moderate level of 6mA (Fig. 1B and C). The number of 6mA sites in genes increased with gene length, and they had a significant positive correlation (R^2^ = 0.72 and *p* < 1.00×10^-22^ for *A. veronii*; Fig. 1D). The vast majority of 6mA sites were located in protein-coding genes, constituting 92.78% of the total distribution, displaying an average of 14.67 6mA sites per gene (Fig. 2A). Conversely, their presence was markedly lower in non-coding RNA genes (0.41%) and intergenic regions (6.81%) (Fig. 2A). The average counts of 6mA sites in non-coding RNA gene and intergenic region were displayed as 1.85 and 1.63, respectively.

The non-coding RNA genes were further divided into rRNA genes, tRNA genes, and sRNA genes (Fig. 2A). The percentages of 6mA sites in the three types of genes were compared. The results showed that the percentage of 6mA sites in tRNA genes (17.70%) was significantly lower than that in rRNA genes (80.66%) (Binomial test, p = 6.99 × 10^–12^), differing by nearly 5 times (Fig. 2A). Similarly, the percentage of 6mA sites in rRNA genes (80.66%) was 50 times higher than that in sRNA genes (1.64%) (Binomial test, p = 1.00 × 10^–22^; Fig. 2A). The mean number of 6mA sites in each rRNA gene was 7.94. In contrast, the average counts for 6mA sites in tRNA and sRNA genes were notably lower, recording at 0.44 and 0.42, respectively. These results indicated that 6mA tended to occur in rRNA genes rather than tRNA genes or sRNA genes. Furthermore, the percentages of protein-coding genes, tRNA genes, rRNA genes, and sRNA genes containing 6mA sites in the entire genome were calculated (Fig. 2B). The results showed that over 90% of protein-coding genes contained 6mA, while rRNA genes were fully methylated. In contrast, the percentages of methylated tRNA genes and sRNA genes were relatively low, only 23.77% and 25.00%, respectively (Fig. 2B). These results indicated that 6mA was widely distributed in *A. veronii*.

To further investigate the distribution characteristics of 6mA, we initially normalized the relative position of each base by dividing it by the total length of a fragment. Subsequently, the frequency of 6mA sites at each relative position was calculated for protein coding genes, non-coding RNA genes and intergenic regions. The results showed that 6mA was more frequently located at 3’ end of protein-coding genes (6.75%), while the frequency of 6mA sites at other positions was only 1.46% on average (Fig. 2C). The frequencies of 6mA sites in different stop codons were compared. TGA was the most frequently used and modified stop codon, while TAG and TAA were not modified by 6mA (Fig. 2D). In non-coding RNA genes, 6mA was found to be concentrated at a limited number of relative positions, with the majority of relative positions not containing 6mA (Fig. 2E). The frequencies of 6mA sites at these relative positions showed significant fluctuations, with the highest frequency reaching as high as 11.52%. Compared to intragenic regions, intergenic regions exhibited diminished frequencies of 6mA sites, averaging merely 0.16% (Fig. 2F). Overall, the distribution of 6mA in intergenic regions tended to be proximal to the gene body (Fig. 2G). Furthermore, we examined the upstream 50 bp of transcription start site (TSS), where the frequencies 46 bp, -42 bp, -37 bp, -31 bp, -17 p, -7 bp, -6 bp, -5 bp were higher compared to other positions (Fig. 2H).

### Effect of 6 mA on gene expression and function in *A. veronii*

**Fig. 3:**
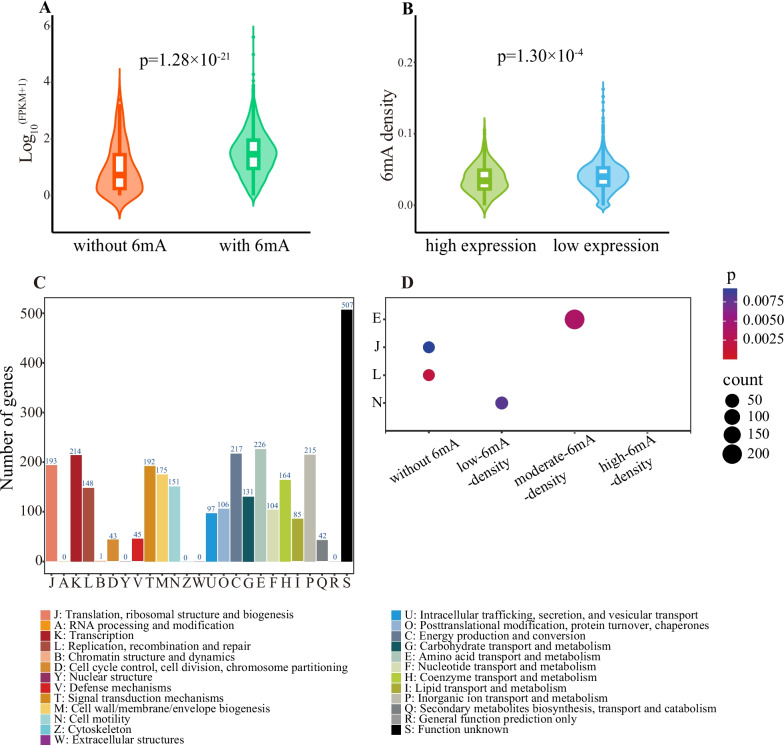
Relationship between 6mA and gene expression or function in *A. veronii*. **A** Comparison of expression levels between the genes without 6mA and those with 6mA. **B** Comparison of 6mA densities between the genes with high and low expression. In both (A) and (B), *P* value was calculated using Wilcoxon test. **C** COG function annotations of protein-coding genes. **D** COG enrichment analyses of protein-coding genes with varying 6mA levels. *P* value was calculated using Fisher’s exact test. The COG categories were presented with false discovery rate-adjusted *P* value < 0.01

Protein-coding genes were divided into genes with 6mA and those without 6mA. Compared to genes without 6mA, those with 6mA had significantly higher expression levels (Wilcoxon test, p = 1.28 × 10^–21^; Fig. 3A), indicating that 6mA had a promoting effect on gene expression. Further comparison of gene expression levels among high-6mA-density, moderate-6mA-density and low-6mA-density genes revealed no significant difference. Upon comparing 6mA levels between genes with high and low expression, the result showed a statistically significant elevation in 6mA levels for genes with high expression in comparison to genes with low expression (Wilcoxon test, p = 1.30 × 10^–4^; Fig. 3B). Finally, the expression levels of protein-coding genes with and without 6mA in the upstream 50 bp of TSS were compared, but no significant difference was found between the two types of genes.

We performed COG functional annotation on protein-coding genes. The results showed that the most abundant functional categories were [K] transcription, [C] energy production and conversion, [E] amino acid transport and metabolism, and [P] inorganic ion transport and metabolism (Fig. 3C). Functional enrichment analysis revealed that genes without 6mA were significantly enriched in [L] replication, recombination, and repair and [J] translation, ribosomal structure and biogenesis (Fisher’s exact test, p = 1.81 × 10^–3^ for [L] and p = 9.26 × 10^–3^ for [J]; Fig. 3D). The low-6mA-density genes were significantly enriched in [N] cell motility (Fisher’s exact test, p = 8.01 × 10^–3^; Fig. 3D) and the moderate-6mA-density genes were significantly enriched in [E] amino acid transport and metabolism (Fisher’s exact test, p = 3.89 × 10^–3^; Fig. 3D). However, the high-6mA-density genes had no significantly enriched functional category.

### Distribution of 6 mA in *H. pylori* genome

**Fig. 4:**
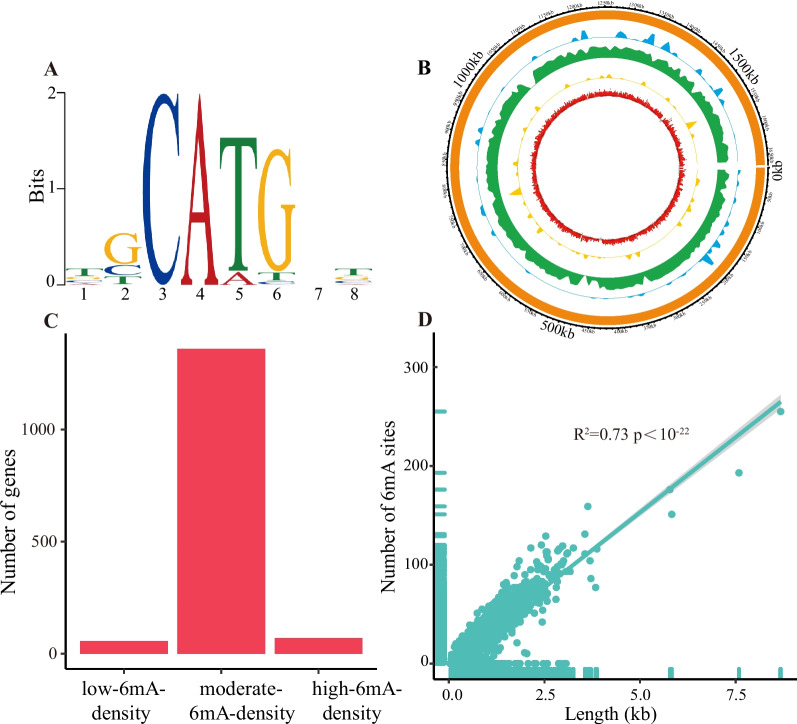
Identification of 6mA in *H. pylori geno*me. **A** The motif significantly enriched with 6 mA. Information content at a motif position, where all nucleoside with equal probability, was 0 bits, while a position with exclusive occurrence of a single nucleotide had an information content of 2 bits. **B** Circos plot illustrating the genomic distribution of 6mA. Blue, green and yellow rings represented the regions with high, moderate and low 6mA levels, respectively. Red ring represented gene expression level. **C** Number of genes with different 6mA levels. The genes were divided into low-6mA-density, moderate-6mA-density and high-6mA-density genes according to their 6mA density. **D** Correlation between the number of 6mA sites and the gene length

For *H. pylori*, both SMRT sequencing and RNA-seq data were available. The identification of 6mA sites was based on SMRT sequencing data, resulting in a total of 49,286 identified 6mA sites, with a corresponding 6mA density (6mA/A) of 4.83% (Table 1). The CATG motif exhibited significant enrichment with 6mA (E-value < 1.00×10^-22^; Fig. 4A), modified by the methyltransferase (GenBank accession number: AAD08252). In particular, a total of 6,191 (83.76%) and 6,216 (84.10%) A bases within the CATG motif were methylated on the positive and negative strands, respectively.

**Fig. 5:**
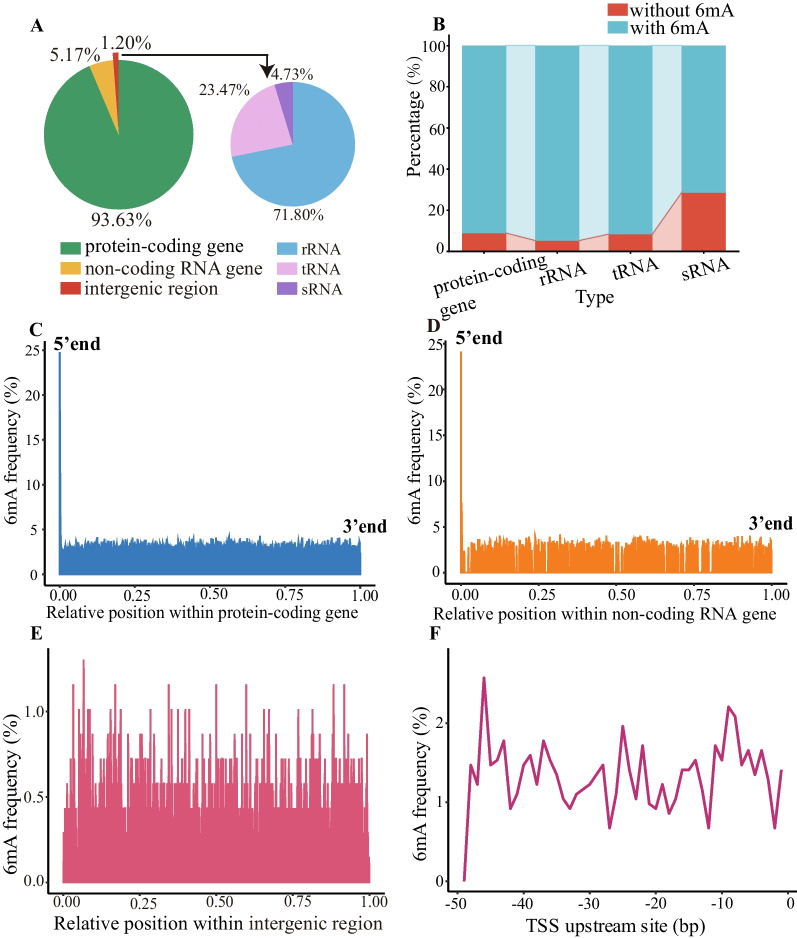
Distribution of 6mA in *H. pylori* genome. **A** Percentage of 6mA sites across different gene types. Each color corresponded to a specific gene type. **B** Percentage of genes with and without 6mA. **C** Frequency of 6mA sites at each relative position within protein-coding genes. **D** Frequency of 6mA sites at each relative position within non-coding RNA genes. **E** Frequency of 6mA sites at each relative position within intergenic regions. In (C-E), if a gene/intergenic region had N bases, the relative position of the base at the i-th position was represented as i/N, ranging between 0 and 1. **F** Frequency of 6mA sites at each position in the upstream 50 bp of transcription start site (TSS)

There were 1566 protein-coding genes and 62 non-coding RNA genes in *H. pylori*. The majority of genes was categorized as the moderate-6mA-density genes (Fig. 4B and C). A positive correlation was observed between gene length and the number of 6mA sites within the gene (R^2^ = 0.73 and *p* < 1.00×10^-22^; Fig. 4D). Protein-coding genes exhibited a significantly higher proportion (93.63%) of 6mA sites compared to non-coding RNA genes (1.20%) or intergenic regions (5.17%) (Fig. 5A). On average, each protein-coding gene contained 29.63 6mA sites, while non-coding RNA gene exhibited an average of 20.89 6mA sites. Intergenic regions displayed a comparatively lower number of 6mA sites with an average of 3.68. When distinguishing among various non-coding RNA genes, it was evident that 6mA predominantly occurred in rRNA genes (71.80%) as opposed to tRNA genes (23.47%) or sRNA genes (4.73%) (Fig. 5A). On average, each rRNA gene contained 59.68 6mA sites. In contrast, tRNA genes and sRNA genes had lower averages of 3.72 and 3.86 6mA sites, respectively. Over 90% of all gene types were found to have 6mA, with the exception of sRNA genes, where only about 70% were modified by 6mA (Fig. 5B).

In protein-coding genes, 6mA exhibited a preference at the 5’ end, with a high frequency of 24.78%, compared to mere 2.36% at the 3’ end (Fig. 5C). Similarly, in non-coding RNA genes, 6mA tended to occur at 5’ end (Fig. 5D). Notably, 26.20% of ATGs in genes were modified by 6mA. In the examination of intergenic regions, the frequencies of 6mA sites were lower, averaging only 0.37% (Fig. 5E). In the upstream 50 bp of TSS, specific positions, namely -46 bp, -43 bp, -37 bp, -25 bp, -22 bp, -11bp, -9 bp, -8 bp endowed with higher frequencies of 6mA sites compared to other positions (Fig. 5F).

### Effect of 6 mA on gene expression and function in *H. pylori*

**Fig. 6:**
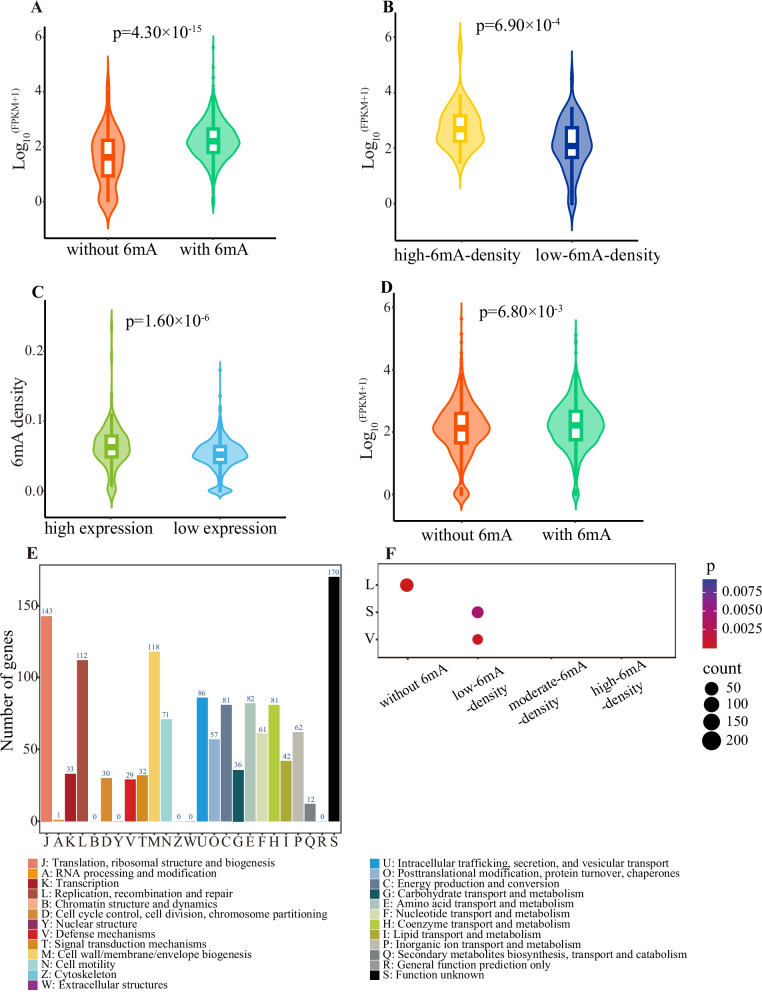
Relationship between 6mA and gene expression or function in *H. pylori*. **A** Comparison of expression levels between the genes without 6mA and those with 6mA. **B** Comparison of expression levels between the genes with high and low 6mA density. **C** Comparison of 6mA densities between the genes with high and low expression. **D** Comparison of expression levels between the protein-coding genes with and without 6mA in the upstream 50 bp of transcription start site. In (A-D), *P* value was calculated using Wilcoxon test. **E** COG function annotations of protein-coding genes. **F** COG enrichment analyses of protein-coding genes with varying 6mA levels. *P* value was calculated using Fisher’s exact test. The COG categories were presented with the false discovery rate-adjusted *P* value < 0.01

Compared to genes without 6mA, those with 6mA exhibited significantly higher expression levels in *H. pylori* (Wilcoxon test, p = 4.30 × 10^–15^; Fig. 6A). Notably, high-6mA-density genes displayed significantly higher expression levels than low-6mA-density genes (Wilcoxon test, p = 6.90 × 10^–4^; Fig. 6B). Furthermore, genes with high expression also had significantly increased 6mA levels compared to genes with low expression (Wilcoxon test, p = 1.60 × 10^–6^; Fig. 6C). A comparison of protein-coding genes with and without 6mA in the upstream 50 bp of TSS revealed that the former exhibited significantly higher expression levels (Wilcoxon test, p = 6.80 × 10^–3^; Fig. 6D).

The COG functions of protein-coding genes were subsequently annotated. The results showed that the majority of genes were associate with [J] translation, ribosomal structure and biogenesis, [M] cell wall/membrane/envelope biogenesis, and [L] replication, recombination, and repair (Fig. 6E). Of which, genes without 6mA were significantly enriched in [L] replication, recombination, and repair (p = 4.50 × 10^–12^; Fig. 6F), while low-6mA-density genes displayed significantly enrichment in [V] defense mechanisms (Fisher’s exact test, p = 4.46 × 10^–4^ for *H. pylori*; Fig. 6F). However, high-6mA-density and moderate-6mA-density genes did not reveal significant enrichment in any functional category.

## Discussion

A. *veronii* and *H*. *pylori* are both pathogenic bacteria in the phylum Proteobacteria [20, 21]. The 6mA densities in their genomes were 3.65% and 4.83% respectively, which were similar to the 6mA densities in other prokaryotic genomes, such as *Xanthomonas campestris* (0.17-3.8%) [22], *Escherichia coli* (1.9%) [23], *Campylobacter coli* (2.7%) [16], and *Pseudomonas aeruginosa* (2.1-4.2%) [24]. However, they were higher than the 6mA densities in eukaryotic genomes, such as *Homo sapiens* (0.083%), *Arabidopsis thaliana* (0.099%), *Caenorhabditis elegans* (0.027%), *Drosophila melanogaster* (0.023%) [25], and *Chlamydomonas reinhardtii* (0.4%) [26]. These results further demonstrated that 6mA was more common in prokaryotes than in eukaryotes [4]. The reason may be that prokaryotes typically have small genomes with higher gene density, while eukaryotes have larger genomes containing noncoding regions. Meanwhile, prokaryotes usually lack histone structures and DNA repair mechanisms, and thus the large number of 6mA sites in prokaryotes may play an important role in maintaining genome integrity and stability[27]. GATC and CATG were the motifs enriched with 6mA sites in *A. veronii* and *H. pylori*. These motifs have also been reported in other species, including *E. coli*, *Xanthomonas oryzae pv. oryzicola*, and *P. aeruginosa* for GATC [16, 22, 23], as well as in *Chlamydomonas* for CATG [26]. The results suggested that the existence of methyltransferases in these species that were accountable for modifying these specific sites. The conserved motifs across diverse species further supported the significance of these methylation patterns in various organisms.

6mA may play different roles in different bacteria, presenting a variety of functions [25]. In *A. veronii*, 6mA was prevalent at 3’ end of protein-coding genes, indicating that a potential functional role of 6mA in the transcription termination. In *H. pylori*, 6mA was predominantly appeared at 5’ end of protein-coding genes, suggesting that a potential involvement of 6mA in the transcriptional initiation. This result may be attributed to the fact that the start codon ATG is part of the specific recognition sequence of the CATG methyltransferase. Notably, the methyltransferase demonstrated high specificity and efficiency [28], with nearly 100% of A bases in the GATC motif and over 80% of A bases in the CATG motif being methylated in this study. Such specificity and efficiency are crucial in regulating various cellular processes, including gene expression, DNA replication, and DNA repair. It has been found that 6mA enriched near TSS could direct the precise localization of RNA polymerase to activate transcription [10, 11, 26, 29]. Compared to the stop codons TAG and TAA, 6mA tended to occur more frequently in TGA of *A. veronii*. Similar result has also been reported in *Xanthomonas oryzae* pv. *Oryzicola* [24]. The frequency of 6mA sites in the upstream 50 bp of TSS presented a trend of first decreasing and then increasing in *H. pylori*, which was similar to the result in *Chlamydomonas* [26].

In *A. veronii*, genes with 6mA had significantly higher expression levels than genes without 6mA, but genes with low expression had significantly higher 6mA levels. This suggested that a certain amount of 6mA in *A. veronii* could promote gene expression, but excessive modification may inhibit gene expression. In some cases, 6mA can prevent specific transcription factors from binding to the promoter region of a gene, thereby reducing gene transcription [30]. This protective effect may help regulate gene expression homeostasis in bacteria [31]. In *H. pylori,* genes with high expression had significantly higher 6mA levels, presenting a positive correlation. These findings provided strong evidence that 6mA promoted gene expression in *H. pylori* and a higher 6mA density may lead to a stronger promoting effect. Similar result has also been reported in *Fragaria vesca* [12]. In this study, the RNA extraction from *A. veronii* cultured in M9 medium was performed during stationary phase using phenol–chloroform. Conversely, for *H. pylori* grown in Brain–heart infusion (BHI) broth, RNA extraction was conducted during exponential phase using TRIzol reagent. The discrepancies in growth stage, medium composition and RNA extraction protocol between the two strains may account for the observed divergent regulatory effects of 6mA on gene expression. Due to the acidity and peristalsis, the stomach has been known to be unfavorable for bacterial survival and colonization [32]. However, *H. pylori* has evolved a defense mechanism that enables it to survive in the stomach, which may be associated with low-6mA-density genes. Because our result showed that low-6mA-density genes were significantly enriched in [V] defense mechanisms. In the follow-up work, we will carry out experimental verification. On the whole, the results of this study provides new insights into the DNA methylation in *A. veronii* and *H. pylori*.

## Conclusions

This study aimed to to explore the distribution characteristics of 6mA at the whole-genome level in *A. veronii* and *H. pylori* and its association with gene expression and function. The results showed that in *A. veronii,* 6mA tended to be localized at the 3’ end of protein-coding genes, with an association with cell motility. Excessive 6mA demonstrated a discernible inhibitory impact on gene expression*.* While in *H. pylori*, 6mA was predominantly situated at 5’ end of protein-coding genes, correlating with defense mechanism. Notably, 6mA exhibited a significant promotional effect on gene expression. In both strains, a higher prevalence of 6mA was observed in rRNA genes compared to tRNA or sRNA genes. These findings shed light on the epigenetic regulation and functional characteristics of *A. veronii* and *H. pylori*. The results contribute to our understanding of bacterial epigenetics and provide valuable insights for future research in this field.

## Materials and methods

### Identification of 6 mA

Raw SMRT sequencing data of *A. veronii* was obtained from our previous study (accession number: SRP457255) [33]. Corresponding data of *H. pylori* was downloaded from the NCBI Sequence Read Archive (SRA) database (accession number: SRP091719). The PacBio SMRT analysis platform (version 6.0.0) (https://www.pacb.com/products-and-services/analytical-software/smrt-analysis/) was used to detect 6mA sites in *A. veronii* and *H. pylori* genomes (Additional file 1). First, the raw sequencing data in h5 format was converted to bam format using the bax2bam command and an index of the reference genome was built using the Samtools command. Then, the bam format data was filtered and aligned to the reference genome using the Pbalign command with the parameters: "–bestn 10 –minMatch 12 –maxMatch 30 –nproc 8 –minSubreadLength 50 –minAlnLength 50 –minPctSimilarity 70 –minPatAccuracy 70 –hitPolicy randombest –randomSeed 1". Furthermore, the 6mA site was identified using ipdSummary with the parameters "–identify m6A –csv h5 kinetics.h5". Finally, the 6mA site with more than 25-fold coverage was used to further analysis (Additional file 2).

### Analysis of 6 mA

For each 6mA site, four bases from its upstream and downstream sequences were extracted respectively [11]. MEME (version 5.4.1) [34] was used to identify conserved motifs in the flanking regions. The gene with 6mA were divided into three groups according to the 6mA density: high-6mA-density genes (K > 1), moderate-6mA-density genes (− 1 ≤ K ≤ 1), and low-6mA-density genes (K <  − 1) [12]. K was calculated according to the following formulas:

$$\text{K } = {\text{log}}_{2}{\text{FC}}$$

$$\text{FC } = \frac{\text{6mA density of a gene}}{\text{6mA density of the genome}}$$

$${6}\text{mA density } = \frac{\text{the number of} \, \text{6mA sites}}{\text{the number of} \, \text{A bases}}$$

To evaluate the distribution characteristics of 6mA, the relative position of each base was computed by dividing the total length of a fragment. If a region comprised N bases, the relative position of the base at the i-th position was represented as i/N, ranging between 0 and 1. We conducted a comparison of the average frequency of 6mA sites at relative positions among protein-coding genes, non-coding RNA genes, and intergenic regions.

### Identification of methyltransferases

The CATG-specific DNA methyltransferase in *H. pylori* has been stored in the REBASE database [35]. To identify the methyltransferase responsible for modifying the GATC motif in *A. veronii,* the sequences of 162 high-quality GATC-specific DNA methyltransferases were downloaded from the REBASE database [35]. Subsequently, a multiple sequence alignment was performed using MUSCLE [36] and a HMM was constructed using the hmmbuild function from the HMMER v3.3.2 suite [37]. The HMM was then aligned with all protein sequences in *A. veronii* using the hmmsearch function from the HMMER v3.3.2 suite [37].

### Analysis of gene expression

Gene expression data of *A. veronii* was obtained from our previous study (accession number: SRP162819) [38]. Raw RNA-seq data of *H. pylori* was downloaded from the NCBI SRA database (accession number: SRP098564). Low-quality data was removed from the raw data using picard (version 1.119) (http://broadinstitute.github.io/picard/). The index of *H. pylori* reference genome was built using bowtie2 (version 2.2.2) [39]. High-quality reads were aligned to *H. pylori* reference genome using Tophat (version 2.1.1) [40]. The gene expression level, i.e., fragments per kilobase of transcript per million mapped reads (FPKM) value, was estimated for *H. pylori* using Cufflinks (version 2.2.1) [10]. If the FPKM value of a gene was greater than the mean FPKM value of all genes, it would be defined as a gene with high expression, otherwise, it would be defined as a gene with low expression. A gene was categorized as exhibiting high expression if its FPKM value surpassed the mean FPKM value of all genes; conversely, it was designated as a gene with low expression if its FPKM value was equal to or less than the mean FPKM value of all genes. Wilcoxon test was used to evaluate the significance of differences, and the result was visualized using the Violin Group tool in Hiplot (ORG) [41].

### COG functional annotation

Protein sequences of *A. veronii* were input into EggNOG (version 5.0) [42] for functional annotation, and the results were output to the OmicShare tools platform (https://www.omicshare.com/tools) for visualization. Additionally, we used R 4.2.2 to perform statistical analysis. Fisher’s test was used for functional enrichment analysis, and the result was visualized using the Dotplot tool [43].

### Supplementary Information

**Additional file 1.** The commands for processing SMRT sequencing data.

**Additional file 2. **The number of 6mA sites.

## Data Availability

The original contributions presented in the study are included in the article, further inquiries can be directed to the corresponding authors.
